# Variation in cost and performance of routine immunisation service delivery in India

**DOI:** 10.1136/bmjgh-2018-000794

**Published:** 2018-06-22

**Authors:** Susmita Chatterjee, Palash Das, Aditi Nigam, Arindam Nandi, Logan Brenzel, Arindam Ray, Pradeep Haldar, Mahesh Kumar Aggarwal, Ramanan Laxminarayan

**Affiliations:** 1 Public Health Foundation of India, Gurgaon, India; 2 Center for Disease Dynamics, Economics & Policy, Washington, District of Columbia, USA; 3 Bill & Melinda Gates Foundation, Washington, District of Columbia, USA; 4 Bill & Melinda Gates Foundation, New Delhi, India; 5 Immunization Division, Ministry of Health and Family Welfare, Government of India, New Delhi, India; 6 Princeton Environmental Institute, Princeton University, Princeton, New Jersey, USA

**Keywords:** health economics, immunisation

## Abstract

A comprehensive understanding of the costs of routine vaccine delivery is essential for planning, budgeting and sustaining India’s Universal Immunisation Programme. India currently allocates approximately US$25 per child for vaccines and operational costs. This budget is prepared based on historical expenditure data as information on cost is not available. This study estimated the cost of routine immunisation services based on a stratified, random sample of 255 public health facilities from 24 districts across seven states—Bihar, Gujarat, Kerala, Meghalaya, Punjab, Uttar Pradesh and West Bengal. The economic cost for the fiscal year 2013–2014 was measured by adapting an internationally accepted approach for the Indian context. Programme costs included the value of personnel, vaccines, transport, maintenance, training, cold chain equipment, building and other recurrent costs. The weighted average national level cost per dose delivered was US$2.29 including vaccine costs, and the cost per child vaccinated with the third dose of diphtheria–pertussis–tetanus (DPT) vaccine (a proxy for full immunisation) was US$31.67 (at 2017 prices). There was wide variation in the weighted average state-level cost per dose delivered inclusive of vaccine costs (US$1.38 to US$2.93) and, for the cost per DTP3 vaccinated child (US$20.08 to US$34.81). Lower costs were incurred by facilities and districts that provided the largest number of doses of vaccine. Out of the total cost, the highest amount (57%) was spent on personnel. This costing study, the most comprehensive conducted to date in India, provides evidence, which should help improve planning and budgeting for the national programme. The budget generally considers financial costs, while this study focused on economic costs. For using this study’s results for planning and budgeting, the collected data can be used to extract the relevant financial costs. Variation in cost per dose and doses administered across facilities, districts and states need to be further investigated to understand the drivers of cost and measure the efficiency of service delivery.

**Key questions**

**What is already known?**

- A comprehensive understanding of the costs of routine vaccine delivery is essential for planning, budgeting and sustaining Universal Immunisation Programme in India.
- Information on the cost of vaccination is not available in India.

**What are the new findings?**

- Personnel cost represented the largest share of total immunisation cost at the facility level followed by vaccines and supplies and incentives for accredited social health activist (ASHA) workers.
- Unit costs (cost per dose delivered, cost per fully immunised child) varied widely across facilities, districts and states.

**What do the new findings imply?**

- Facility-level unit cost information can be used by the district and sub-district level officials for identifying inefficiencies.
- Aggregated (district, state and national level) information is useful for programme planning and budgeting at each level.

## Introduction

India’s Expanded Programme on Immunisation, introduced in 1978, provided vaccines to protect children against diphtheria, pertussis and tetanus (DPT), poliomyelitis (OPV), tuberculosis (BCG) and typhoid-paratyphoid [1]. The initiative was expanded under the Universal Immunisation Programme (UIP) in the mid-1980s to include tetanus toxoid vaccine for pregnant women in 1983 and measles in 1985.^1^ Currently, the programme includes BCG, hepatitis B, OPV, DPT, measles, *Haemophilus influenzae* type B (Hib) containing pentavalent (DPT+Hepatitis B+Hib), inactivated polio vaccine (IPV), Japanese encephalitis (JE in endemic districts) and tetanus toxoid (TT) vaccines. Rotavirus vaccine has been introduced in nine states, and pneumococcal vaccine has recently been introduced in some cohorts of children in three states with a plan to rapidly scale up in other cohorts and states.

India’s UIP is the largest immunisation programme in the world, aiming to administer all primary vaccines to 26 million newborns each year through 9 million immunisation sessions, which are primarily in the form of outreach sessions at the village level.^2^ A total of 289 million vaccine doses were administered in 2016–2017.^3^

Understanding the total cost, and its variation and breakdown, is important for national immunisation programmes as they plan and budget for delivering services and introducing new vaccines. In India, immunisation budgets at the central and state levels are prepared based on historical expenditure data as information on cost is not available. This study fills the knowledge gap by estimating the economic cost of routine immunisation programme at health facility, district, state and national levels.

## Methods

Routine immunisation services in India are delivered at health facilities and through outreach activities and special immunisation weeks. This study collected data to estimate the costs of these delivery mechanisms but did not include the cost of supplementary immunisation activities and outbreak response. Cost data were retrospectively collected by trained enumerators using standardised and pre-tested questionnaires for the fiscal year 2013–2014 over a period of a year in 2014–2015. All costs were calculated for April 2013 to March 2014 and converted into 2017 US dollars. An average exchange rate of 2017, US$1=INR 64, is used throughout the paper.

### Sampling methodology

To generate nationally representative estimates, this study relied on a stratified, random sampling design. India’s 29 states were stratified into six levels of development (level 1 represented the most developed and level 6 the least developed) using the following state-level indicators: (a) infant mortality rate, (b) female literacy rate, (c) full immunisation coverage rate and (d) per capita income.^4–7^ Within each level of development, states were further classified into different regions. One state from each level of development was deliberately selected so that all six geographic regions of the country were represented. The online supplementary table A1 presents the classification of states according to levels of development and geographical regions.

10.1136/bmjgh-2018-000794.supp1 Supplementary file 1

Districts within each state were ranked into three or four strata based on the scores obtained from the following district level indicators: number of children aged 0–6 years, proportion of households living in rural areas, proportion of children aged 0–6 years receiving full immunisation and number of health facilities per 1000 children.^5 8 9^ These variables were intended to capture the variation in demand and utilisation of immunisation services at the facility level. One district from each stratum was randomly selected using computer application that employs random number generator, resulting in three to four districts per state in the sample.

Within each district, two blocks (sub-districts) were purposively selected based on two indicators: percentage of socioeconomically disadvantaged groups (known as scheduled caste or scheduled tribe) in the population and female literacy rate. The blocks were selected to cover the lower and upper extremes of these indicators. These variables are known to be related to demand and utilisation of health services.^10^Immunisation services are provided in the Post-Partum Unit (PP) in district hospitals. The PP units provide all routine vaccines generally in fixed session sites at the facilities and these were included in the sample in the study.

Each block typically has one Community Health Centre (CHC, a 30-bed hospital or referral unit with specialised services), which was included in this sample.^11^ In addition, two or three Primary Health Centres (PHCs) associated with the CHC as well as one to two subcentres (SCs) associated with each selected PHC were randomly selected. SCs are the first contact point between the primary healthcare system and the community, and each PHC is a four-bed to six-bed referral unit for six SCs.

The final sample consisted of 99 SCs, 89 PHCs, 44 CHCs and 23 PP units in 24 districts of seven states of India: Bihar, Gujarat, Kerala, Meghalaya, Punjab, Uttar Pradesh and West Bengal. Table 1 illustrates the final study sample.

**Table 1:** Study sample

State	Districts	Sub-districts (blocks)	Health Facilities per State
Bihar	Aurangabad East Champaran Jehanabad	Aurangabad Sadar Goh Madhuban Motihari Sadar Jehanabad Makhdumpur	Post-Partum Units: 3 Community Health Centres: 5 Primary Health Centres: 7 Sub-centres: 9 **Total: 24**
Gujarat	Banaskantha Gandhinagar Rajkot Tapi	Amirgadh Palanpur Dehgam Gandhinagar Jasdan Rajkot Uchchhal Vyara	Post-Partum Units: 4 Community Health Centres: 6 Primary Health Centres: 18 Sub-centres: 20 **Total: 48**
Kerala	Alappuzha Kozhikode Pathnamthitta	Alappuzha Chenganur Kozikode Vadakara Kozhencherry Ranny	Post-Partum Units: 3 Community Health Centres: 6 Primary Health Centres: 12 Sub-centres: –12 **Total: 33**
Meghalaya	East Khasi Hills Jaintia Hills West Khasi Hills	Mawkenrew Mylliem Amlarem Laskein Mairang Mawshynrut	Post-Partum Units: 3 Community Health Centres: 5 Primary Health Centres: 13 Sub-centres: 9 **Total: 30**
Punjab	Amritsar Muktsar Rupnagar Sangrur	Ajnala Amritsar II Gidderbaha Muktsar Anandpur Sahib Rupnagar Lehra Sangrur	Post-Partum Units: 4 Community Health Centres: 8 Primary Health Centres: 16 Sub-centres: 20 **Total: 48**
Uttar Pradesh	Etah Lucknow Muzaffarnagar	Etah Jalesar Malihabad Sarojini nagar Jansath Muzaffarnagar	Post-Partum Units: 2 Community Health Centres: 6 Primary Health Centres: 6 Sub-centres: 10 **Total: 24**
West Bengal	Bankura Howrah Malda North 24 Parganas	Bankura II Ranibandh Bally Jagaccha Uluberia I English Bazar Habibpur Barasat I Sandeshkhali I	Post-Partum Units: 4 Community Health Centres: 8 Primary Health Centres: 17 Sub-centres: 19 **Total: 48**
**Total: 7**	**24**	**48**	**255**

### Costing methodology

A government provider perspective was used for the study. Economic costs of the routine immunisation programme were calculated based on an internationally recognised and standardised approach, adapted to the Indian context.^12 13^ While financial cost focuses on the financial outlays related to the programme, economic costs represent the opportunity costs associated with the programme as compared with their next best alternatives and include valuation of all inputs needed for the programme including valuation of time, supplies, equipment and annualisation of costs that adjusts for a discount rate.^12^ The main cost categories included in this analysis were personnel, vaccines and supplies, travel and transport, training, maintenance and overhead expenses, incentives and the annual value of capital expenditures, such as cold chain, building and vehicles. Data were gathered from financial reports, monthly reports on immunisation, immunisation registers showing the total vaccines administered by vaccinators and vaccine stock and issue registers. District, state and national level data were gathered from the respective administrative head offices.

At the facility level, personnel costs were calculated based on the salary and allowances for staff involved in immunisation delivery (physicians, auxiliary nurse midwives, lady health volunteers, cold chain handlers, among others), and estimates of the time spent for administering or transporting vaccines, record-keeping and travel to immunisation sessions for 285 days per year. Average annual gross salary of different categories of staff is given in table 2. Trained enumerators interviewed different categories of staff to determine person-time spent on immunisation activities.

**Table 2:** Average salary of different categories of staff related to immunisation (US$ 2017)

Immunisation related staff	Annual gross salary
State immunisation officer	32 096
State cold chain officer	13 308
State vaccine store in-charge	7922
District immunisation officer	44 091
District vaccine store in-charge	17 035
District cold chain technician	13 614
Driver	7117
Block medical officer	21 934
Cold chain handler at block level	9669
Auxiliary nurse midwife (Regular)	8095
Auxiliary nurse midwife (Contractual)	2151
Lady health volunteer	11 000

Source, Field Survey

Vaccine costs were estimated by multiplying doses used (including wastage) by unit prices of vaccines. The details of vaccines used in India’s immunisation programme during the study period are given in table 3. Wastage rates were calculated at the vaccinator level by subtracting doses administered from doses issued and returned (doses used), divided by doses used. If an open vial policy was applicable, doses issued were not always equal to doses used, and the vaccinators retuned the opened vials if not fully used. Therefore, returned doses were subtracted from doses issued to estimate the used doses. Vaccine wastage rates could only be estimated using this approach for 64% of vaccinators because of data limitations. Wastage rates used in the cost analysis of India’s comprehensive multi-year plan for immunisation (cMYP) were used for the remaining sample of vaccinators.^14^

**Table 3:** Details of vaccine schedule for children under 2 (April 2013 to March 2014)

Vaccines	Recommended doses	Target group	Doses per vial	Price per dose (2013 US$)*	Average wastage†	Study states
Bacillus Calmette Guerin (BCG)	1	At birth or as early as possible till 1 year of age	10	0.05	56%	Bihar, Gujarat, Kerala, Meghalaya, Punjab, Uttar Pradesh, West Bengal
Hepatitis B birth dose	1	At birth or as early as possible within 24 hours	10	0.06	30%	Bihar, Gujarat, Kerala, Meghalaya, Punjab, Uttar Pradesh, West Bengal
Oral polio vaccine (OPV) zero dose	1	At birth or as early as possible within first 15 days	20	0.08	36%	Bihar, Gujarat, Kerala, Meghalaya, Punjab, Uttar Pradesh, West Bengal
Hepatitis B	3	At 6 weeks, 10 weeks and 14 weeks	10	0.06	30%	Bihar, Meghalaya, Punjab, Uttar Pradesh, West Bengal
OPV	3	At 6 weeks, 10 weeks and 14 weeks	20	0.08	36%	Bihar, Gujarat, Kerala, Meghalaya, Punjab, Uttar Pradesh, West Bengal
Diphtheria–pertussis–tetanus (DPT)	3	At 6 weeks, 10 weeks and 14 weeks	10	0.06	32%	Bihar, Meghalaya, Punjab, Uttar Pradesh, West Bengal
Hib containing pentavalent	3	At 6 weeks, 10 weeks and 14 weeks	10	2.11	27%	Gujarat, Kerala
Measles 1 st dose	1	9–12 months	5	0.17	37%	Bihar, Gujarat, Kerala, Meghalaya, Punjab, Uttar Pradesh, West Bengal
DPT 1 st booster	1	16–24 months	10	0.06	32%	Bihar, Gujarat, Kerala, Meghalaya, Punjab, Uttar Pradesh, West Bengal
OPV 1 st booster	1	16–24 months	20	0.08	36%	Bihar, Gujarat, Kerala, Meghalaya, Punjab, Uttar Pradesh, West Bengal
Measles second dose	1	16–24 months	5	0.17	37%	Bihar, Gujarat, Kerala, Meghalaya, Punjab, Uttar Pradesh, West Bengal

*Source: Immunisation Division, Ministry of Health and Family Welfare, Government of India, personal communication. †Wastage rates are an average for 164 of the 255 vaccinators.

Cost of supplies such as syringes, paracetamol tablets, plastic bags, immunisation cards and tally sheets were estimated based on the numbers used per child and per session and number of children vaccinated and sessions. The actual expenditures on training, maintenance of cold chain equipment and vehicle, waste management, printing, stationery, transport cost for vaccine delivery, meetings, reporting, travelling to immunisation sessions and incentives during the study period were gathered from the financial records of each sampled facility and were included in the analysis.

Performance-based incentives are given to accredited social health activists (ASHAs) for promoting universal immunisation along with other healthcare programmes. Specifically for immunisation, the ASHAs were paid US$1.6 per child for full immunisation in first year, US$0.8 for a child’s complete immunisation up to age 2 and US$2.3 for child mobilisation per session during the study period. Another payment was made for alternate vaccine delivery (AVD). AVD is a vaccine delivery system introduced by the government to deliver vaccines from cold chain points to the outreach sites on each session day. During the study period, the person responsible for AVD was paid US$1.2 per session and US$2.3 for hard-to-reach areas. Actual expenditure for ASHA incentives for immunisation and AVD during the study period were collected from the financial records of each sampled facility and were factored in the cost analysis.

The annualised discounted costs of cold chain equipment, vehicles and buildings were included in this analysis. Allocation of building space to immunisation was based on the proportion of facility space used for immunisation purposes. Allocation of vehicle was based on the number of days the vehicle was used for immunisation. A 3% discount rate and country-specific useful life-years of capital items was also included in the analysis.^15^ Average price and useful life of cold chain equipment and vehicle are given in table 4. Overhead expense, such as electricity, was allocated to immunisation and cold chain rooms based on a share of facility space used for immunisation.

**Table 4:** Price and useful life of cold chain equipment and vehicle (2017 US$)

Cold chain equipment	Average price (per unit)	Useful life
Walk-in cooler/walk in-freezer (large)	32 610	10
Walk-in cooler/walk in-freezer (small)	27 175	10
Ice-lined refrigerator/deep freezer (large)	906	10
Ice-lined refrigerator/deep freezer (small)	725	10
Solar direct drive refrigerator	3623	10
Stabiliser	91	3
Vaccine carrier	18	3
Cold box (large)	133	5
Cold box (small)	91	5
Ice packs	0.63	2
Vehicle		
Vaccine van	10 909	10
Shared vehicle	14 493	10

Source, Immunisation Division, Ministry of Health and Family Welfare, Government of India, personal communication except for price of shared vehicle which was collected during field survey.

The total facility immunisation cost for routine immunisation in SCs, PHCs, CHCs and PPs was divided by estimates of the target children per facility, number of total doses administered, number of children vaccinated with DPT3 and number of fully immunised children (FIC)^i^ to calculate unit costs, which were compared across facility types: cost per target child, cost per dose, cost per DPT3 child and cost per FIC.

### Aggregation of costs to the district, state and national level

Immunisation programme costs incurred at district and state levels were related to supervision, monitoring, management and maintenance of the supply chain and distribution of vaccines. These costs were estimated using the same approach used at the facility level. To determine overall district- and state-level costs, the weighted average immunisation cost exclusive of vaccine costs by type of facility was multiplied by the number of facilities by type in each district or state. To this figure, the total vaccine costs for the district or state were added. Weights were based on sampling probabilities of selecting facilities, blocks, and districts. A similar procedure was used to aggregate immunisation programme costs to the national level. Central level costs of time spent by officials in the immunisation division of the ministry, operational costs of the government medical store depots and costs of vaccines for the country were added to generate total national level immunisation costs.

## Results

### Immunisation programme outputs

The target population and the number of vaccinated children less than 1 year of age varied widely across locations. While average target children per vaccinator at Kerala SC was 76, the same in Bihar SC was 279. The average DPT1 to DPT3 dropout rate also varied widely across facilities: 3%–12% at SCs, 4%–17% at PHCs and 3%–13% at CHCs. Basic information about the sampled facilities are given in online supplementary table A2.

### Cost profiles at the facility level

Personnel costs represented the largest share of total immunisation costs for all types of facilities in all states in the study except for Gujarat SCs and PP units, where vaccines and supplies had the largest share because of use of pentavalent vaccine (not reported in table). Personnel costs ranged from 30%–64% in SCs, 49%–74% in PHCs, 38%–77% in CHCs and 31%–75% in PP units. Next major cost components were vaccines and supplies (about 18%) and ASHA incentives (about 10%). Capital cost was insignificant compared with recurrent expenses for all types of facilities; however, cost components under capital cost had varied contribution across facilities. While cold chain equipment was a major cost component for PHCs and CHCs, building cost dominated in PP units because of semi-urban and urban locations.

### Unit cost estimates

**Figure 1:**
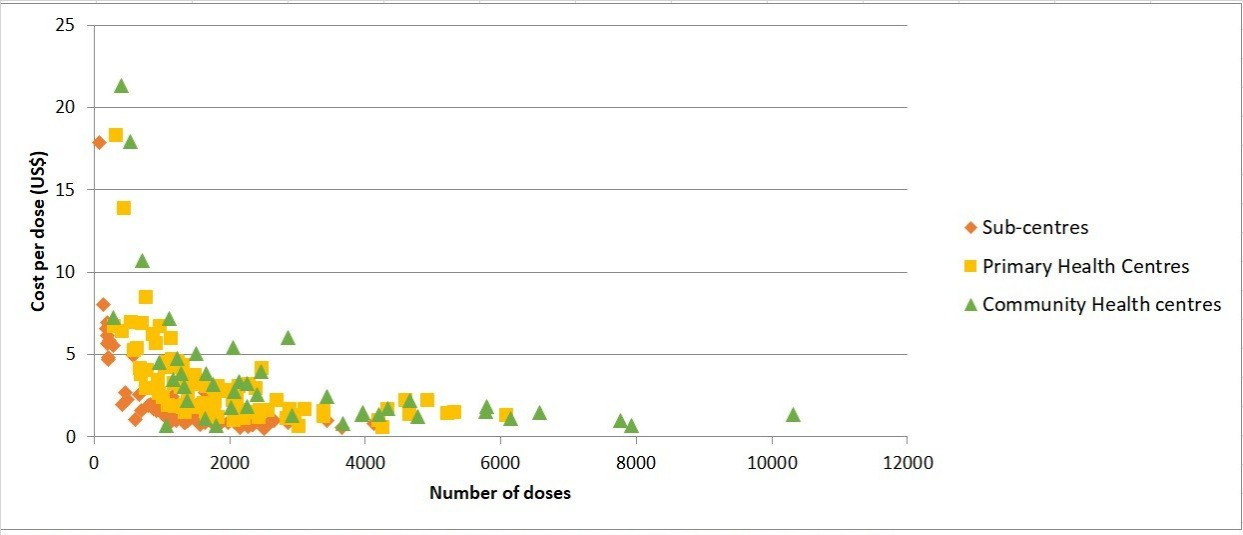
Cost per dose (excluding vaccine cost) versus doses administered (230 facilities) Note: 23 postpartum units were excluded because of their usage of large numbers of doses, compared with smaller facilities. Two CHCs that administered more than 15,000 doses were also excluded.

Figure 1 illustrates the variation in cost per dose plotted against doses administered for different types of facilities. Facility-level unit costs are presented in table 5. Cost per dose, including vaccine cost, was the lowest for district hospital PP units in all states probably because of substantial number of doses administered (online supplementary table A2). The PP units being at the district hospitals generally have higher vaccine load compared to other types of facilities. For other indicators such as cost per DPT3/pentavalent3 child and cost per FIC, no clear trend was visible across facilities. However, the unit costs were generally lower in SCs when compared with PHCs and CHCs for all states except Kerala. Among all study districts, the weighted average cost per dose and the cost per FIC were highest in Banaskantha district in Gujarat (table 6). Cost per DPT3/pentavalent3 child was highest in Tapi district in Gujarat, and second highest in districts in Kerala. Unit costs were highest in Banaskantha district in Gujarat probably because of low immunisation coverage rate (39%) in this district as per the data used for district stratification.^6^ Unit costs, including vaccine costs, were generally higher in the districts of Gujarat and Kerala likely because of usage of pentavalent vaccine in these two states during the study period. Pentavalent vaccine was more expensive than the DPT and hepatitis B vaccines taken together (US$2.11 per dose versus US$0.12 per dose) (table 3). The lowest unit costs per dose and per FIC were for Lucknow district in Uttar Pradesh.

**Table 5:** Average unit cost, including vaccine cost (2017 US$), by facility type

	Bihar	Gujarat	Kerala	Meghalaya	Punjab	Uttar Pradesh	West Bengal
Sub-centre							
Sampled facilities	9	20	12	9	20	10	19
Cost per target child*	12 (7–24)	26 (16–42)	29 (16–50)	20 (11–47)	28 (13–40)	15 (7–22)	26 (15–39)
Cost per dose	1.4 (0.9–3)	2 (1.5–4)	9 (4–22)	2 (1–3)	2 (1–3)	1 (0.8–2)	2 (1–3)
Cost per DPT3/pentavalent3 child	20 (11–49)	27 (16–53)	82 (34–220)	25 (13–44)	32 (18–46)	19 (11–27)	29 (16–44)
Cost per fully immunised child	20 (12–52)	27 (19–52)	74 (31–192)	31 (16–65)	31 (18–43)	17 (9–34)	28 (17–43)
Primary Health Centre							
Sampled facilities	7	18	12	13	16	6	17
Cost per target child*	14 (10–18)	60 (28–94)	138 (64–240)	33 (10–66)	39 (18–99)	19 (18–19)	34 (17–58)
Cost per dose	1.5 (0.9–2)	6 (3–11)	9 (3–22)	3 (1–5)	3 (2–7)	1.8 (1.5–2)	3 (1–5)
Cost per DPT3/pentavalent3 child	21 (12–25)	62 (32–107)	74 (30–186)	45 (12–78)	42 (19–103)	25 (19–29)	36 (17–70)
Cost per fully immunised child	21 (10–28)	65 (31–121)	80 (31–201)	50 (12–88)	42 (20–97)	23 (15–28)	39 (18–68)
Community Health Centre							
Sampled facilities	5	6	6	5	8	6	8
Cost per dose	7 (0.8–25)	3 (2–9)	10 (3–22)	3 (2–4)	3 (1–5)	2 (1–2)	4 (2–6)
Cost per DPT3/pentavalent3 child	98 (15–338)	170 (18–816)	85 (27–180)	41 (26–58)	44 (18–89)	30 (22–50)	60 (35–88)
Cost per fully immunised child	133 (16–483)	169 (16–816)	91 (35–217)	41 (28–55)	48 (17–106)	28 (19–42)	57 (35–88)
District Hospital Post-partum Unit							
Sampled facilities	3	4	3	3	4	2	4
Cost per dose	0.7 (0.4–0.9)	2 (1–5)	1.7 (1.3–2.1)	1 (0.6–2)	0.8 (0.5–1)	0.5 (0.4–0.6)	0.5 (0.4–0.7)
Cost per DPT3/pentavalent3 child	17 (8–22)	47 (30–83)	36 (31–46)	22 (16–26)	19 (7–35)	10 (9–11)	46 (7–107)
Cost per fully immunised child	18 (9–24)	56 (29–111)	43 (35–55)	26 (16–31)	20 (9–38)	10 (8–12)	47 (6–102)

Note: US$1=INR 64.

*Target child is the 0–1 year target of each sampled vaccinator. Figures in parentheses indicate range. Because most vaccinators at CHCs and PP units were unable to provide information on target children, we omitted cost per target child for these facilities.

**Table 6:** Weighted average district-level unit costs, including vaccine cost (2017 US$)

Districts	Cost per dose	Cost per DPT3/pentavalent3 child	Cost per fully immunised child
Bihar			
Aurangabad	2.53	40.45	40.98
East Champaran	1.68	26.10	30.48
Jehanabad	2.83	45.33	47.36
Meghalaya			
East Khasi Hills	1.77	29.27	31.93
Jaintia Hills	2.18	24.93	27.63
West Khasi Hills	2.88	38.75	44.51
Punjab			
Amritsar	1.64	25.02	25.51
Muktsar	2.20	31.70	34.03
Rupnagar	2.17	33.49	34.15
Sangrur	2.20	32.95	33.58
Uttar Pradesh			
Etah	1.32	17.50	33.80
Lucknow	1.01	13.66	11.74
Muzaffarnagar	2.23	25.39	22.69
West Bengal			
Bankura	1.99	31.49	32.79
Howrah	1.71	25.67	27.25
Malda	1.57	23.32	24.98
North 24 Parganas	1.99	29.39	30.23
Gujarat			
Banaskantha	6.18	30.88	68.84
Gandhinagar	1.91	22.92	23.28
Rajkot	2.04	25.41	24.88
Tapi	4.46	55.85	55.52
Kerala			
Alappuzha	4.63	52.16	53.23
Kozhikode	3.08	37.63	39.62
Pathnamthitta	4.71	55.84	57.86

Notes: US$1=INR 64; During the study period, Gujarat and Kerala used pentavalent vaccines while other five states used Diphtheria Pertussis Tetanus vaccine and hepatitis B vaccine.

Cost per dose delivered inclusive of vaccine cost varied from US$1.38 in Bihar to US$2.93 in Kerala (table 7). The cost per FIC was the lowest in Uttar Pradesh (US$18.98) and the highest in Kerala (US$35.50). The generally higher unit costs in Kerala was probably because all immunisation sessions required the presence of a doctor, while in other states, this requirement was not enforced. In addition to the vaccinator and doctor, one or two more staff members (eg, public health nurse) were present in almost all sessions in Kerala, further raising the personnel costs. Weighted average unit costs in Kerala were higher than the national estimates for all indicators (table 7). Cost per target child in Meghalaya and Punjab were higher than the national average, cost per FIC in Meghalaya was higher than the national average and cost per dose delivered in Gujarat was higher than the national average. Total estimated cost of delivering routine immunisation services at the national level was US$737 million at 2017 prices.

**Table 7:** Weighted average state and national level unit costs, including vaccine cost (2017 US$)

	Bihar	Gujarat	Kerala	Meghalaya	Punjab	Uttar Pradesh	West Bengal	National estimate
Cost per target child*	16.43	25.17	35.39	32.07	29.74	17.03	24.39	27.98
Cost per dose	1.38	2.40	2.93	2.09	2.06	1.51	1.68	2.29
Cost per DPT3/pentavalent3 child	20.14	27.92	34.81	29.67	31.27	20.08	22.90	31.67
Cost per fully immunised child	22.03	28.71	35.50	33.30	32.32	18.98	27.32	32.43

Note: US$1=INR 64.

*0–1 target infant for the state and the country.

## Discussion

This paper estimates the costs of delivering routine immunisation services in a large sample of 23 PP units, 44 CHCs, 89 PHCs and 99 SCs in seven states in India. The results are in conformance with those for other countries. For instance, personnel cost is the largest immunisation cost component as observed in this study and the same was found in other studies in Ghana, Honduras, Moldova, Uganda and Zambia.^12 16 17^ This is also in line with a study conducted in the Tamil Nadu state in India, which evaluated immunisation costs and coverage using a longitudinal panel data set collected from 59 health facilities (more than 200 observations) during 1989–1991.^10^

The immunisation cost estimates were generally higher for facilities in Kerala, likely because the way immunisation sessions were planned. The presence of doctors in all immunisation sessions did not reduce the workload at the higher levels and invites the question of whether the policy improves the quality of immunisation services.

Facilities in Kerala also have the highest total immunisation cost per DPT3/pentavalent3 child (US$34.81). The result is within the range of estimates found worldwide^12 16–18^ but is on the lower end, perhaps because of the lower cost of vaccines in India. Local manufacturing and high volumes—most vaccines in India are produced within the country and a large cohort (26 million children) is vaccinated each year—account for the low price. For example, the prices per dose of BCG, pentavalent and OPV vaccines in Zambia were US$0.1, US$3.1, US$0.1, respectively, compared with US$0.05, US$2.1 and US$0.08 in India.^14 19^

The cost estimates in this study are higher than estimates found in the comprehensive multiyear plan (cMYP) of India.^14^ The present study estimated total cost of delivering routine immunisation services at the national level during 2013–2014 at US$737 million, while the government expenditure reported in the cMYP mid-term review during that period was US$636 million (both adjusted at 2017 prices). The lower costs reported in the cMYP stem from an underestimation of the shared costs of buildings and vehicles, the components that were considered in the present study. For example, in the cMYP, it was not possible to consider the space used at the facility level for conducting immunisation sessions or shared vehicles used for transporting vaccines. All these were considered in the present study. The cost per DPT3 child at the national level reported for this study (US$31.67) was much higher than that estimated for the cMYP, US$12.36 (adjusted at 2017 prices).^14^ It should be noted in this context that even though the cMYP costing includes both immunisation specific costs (such as vaccines, per diems) and shared cost, the cMYP does not always discount the shared cost. Therefore, the cMYP estimates and the present study estimates are not strictly comparable.

This study has shown that unit costs at the facility, district and state level vary widely. Using the data from this study, another study examined the determinants of routine immunisation costs in India.^20^ Total facility cost (of SCs and PHCs), excluding the vaccine cost, was the main outcome variable of the regression analysis and the explanatory variables at the facility level were doses administered, type of the facility, distance of the facility from the nearest cold chain point, average salary of the vaccinator, number of immunisation sessions and ratio of third doses of DPT vaccine to total doses administered. Because of measurement error, the analysis did not consider vaccine wastage rate and coverage rate as explanatory variables in the regression. The study found that doses administered, facility type, salary of the main vaccinator and the number of immunisation sessions were significantly associated with the total facility cost excluding vaccine cost.^20^ The study focused only on the determinants of cost at the PHCs and SCs as the CHCs and PP units had different mode of operations in different study states, and therefore, were not added in the regression analysis. Further, the sample size for CHCs and PP units did not allow separate regressions for these facilities.

India’s immunisation programme is mostly funded by the government with some support from immunisation partners and donors.^14^ In the present cost analysis, only Gavi-supported pentavalent vaccines were considered. Subsequently, the government of India has introduced IPV, rotavirus and pneumococcal vaccines with some support from Gavi. As India will be transitioning from Gavi support soon, the government has to take full financial responsibility of the programme to maintain sustainability, which may pose some challenges. Among the study states, per capita immunisation cost compared with per capita public health expenditure ranged between 2.6% in Kerala and Meghalaya to 9% in Bihar. Scaling up immunisation coverage and introducing new vaccines will further increase total financial requirements. The study noted that the major cost component for immunisation is personnel cost. As India has shortage of healthcare workforce, relying heavily on shared personnel may affect the efficient implementation of the programme.

### Study limitations

Costing studies rely on a set of assumptions that help to allocate shared costs between services. Allocation rules for this study have been informed by broader experiences of other field-based studies and international guidance. Because time allocation is based on subjective recall, the value of personnel costs may be either under- or over-estimated. However, through ongoing supervision and review of questionnaires, we were able to address any obvious outliers in terms of time allocation to minimise this bias. Time spent for surveillance activities was not specifically considered because of difficulty in assessing this at the facility level. Immunisation services may share a facility’s administrative staff or guards, whose personnel costs were not considered. A time-motion study was not pursued given the resource constraints related to the large sample size in this study.

## Conclusion

This study of immunisation costing represents one of the most comprehensive exercises done in India at the facility level. We found wide variation in total and unit costs per child. Information from this study on cost per target child can be used to prepare programme budgets at the district, state and national levels. However, not all costs considered in this study are used for preparing budget. Programme budget considers the financial cost, but the main cost component of this study (personnel) is generally not included in the budget. To use this study results for planning and budgeting, one has to extract the relevant financial costs from the data collected. Further, the study findings will help refine the inputs and assumptions for the next five-year cMYP for India. Finally, the economic cost of the programme can be used as input for cost-effectiveness analysis.

This study calculated the actual cost of delivering immunisation services at both fixed and outreach sessions. However, it did not attempt to calculate the cost of reaching India’s less accessible sites. Policy-makers may need different strategies to go the last mile in certain areas, and cost information on outreach sessions in these locations will help the government allocate funding. Even though India’s UIP is more than 30 years old, only 62% of children less than 1 year of age have been reached with life-saving vaccines.^21^ The additional resources required to make coverage truly universal would be worthwhile to estimate.

## Footnotes

i Fully immunised child in India denotes a child aged between 9–11 months who has received BCG, three doses of DPT/pentavalent, three doses of OPV and measles.
