# Supplementary material for: Variation in cost and performance of routine immunisation service delivery in India
Source: BMJ Glob Health. 2018 Jun 22;3(3):e000794. doi: 10.1136/bmjgh-2018-000794 (PMC6014207; doi:10.1136/bmjgh-2018-000794)
Supplement: Supplementary file 1 [file bmjgh-2018-000794supp001.pdf]

## Annexure

### Table A1. States in India, stratified by level of development and region

| | *Level of development* | | | | | |
|---|---|---|---|---|---|---|
| *Region* | I (Most developed) | II | III | IV | V | VI (least developed) |
| North | Delhi | Himachal Pradesh, **Punjab** | Haryana, Uttarakhand | | Jammu and Kashmir | |
| Central | | | | | Chhattisgarh | Madhya Pradesh, **Uttar Pradesh,** Rajasthan |
| East | | | **West Bengal** | | Odisha, Jharkhand | **Bihar** |
| Northeast | Sikkim | Mizoram, Tripura | | Manipur, Nagaland, **Meghalaya** | Arunachal Pradesh | Assam |
| West | Maharashtra, Goa | | | **Gujarat** | | |
| South | **Kerala** | Tamil Nadu | Karnataka | Andhra Pradesh | | |

Note: States in bold were selected for the study

**Table A2. Basic Information for facilities, April 2013–March 2014**

| | Bihar | Gujarat | Kerala | Meghalaya | Punjab | Uttar Pradesh | West Bengal |
|---|---|---|---|---|---|---|---|
| **Sub-centre** | | | | | | | |
| Sampled facilities | 9 | 20 | 12 | 9 | 20 | 10 | 19 |
| Target population | 9644 (6050–14000) | 5987 (3044–15000) | 5743 (3165–9336) | 4048 (2628–6661) | 6755 (4413–11438) | 6811 (4249–9986) | 6246 (3899–12800) |
| Target children (0–1 year) | 279 (182–420) | 123 (42–217) | 76 (31–171) | 129 (72–190) | 121 (64–233) | 199 (115–300) | 104 (40–299) |
| Doses administered | 2357 (1644–3650) | 1364 (417–2537) | 262 (71–663) | 1334 (913–1979) | 1416 (903–3438) | 1988 (1366–2859) | 1379 (458–4134) |
| Sessions conducted (RI) | 120 (90–171) | 48 (24–86) | 18 (12–28) | 81 (52–132) | 62 (45–120) | 80 (63–100) | 40 (23–60) |
| Children vaccinated with DPT3/pentavalent3 | 173 (104–291) | 122 (33–221) | 28 (7–81) | 100 (65–150) | 101 (66–233) | 158 (85–234) | 97 (35–285) |
| Fully immunized children | 171 (104–240) | 116 (39–197) | 35 (8–89) | 85 (41–135) | 105 (66–237) | 170 (86–260) | 97 (36–269) |
| DPT3/pentavalent3 coverage rate (%) | 62 (44–75) | 100 (57–140) | 38 (23–55) | 84 (49–125) | 88 (38–133) | 79 (60–98) | 94 (57–138) |
| Full immunization coverage rate (%) | 62 (41–82) | 97 (61–140) | 44 (21–66) | 73 (22–119) | 90 (42–128) | 87 (45–125) | 95 (68–141) |
| DPT1 to DPT3/pentavalent1 to pentavalent3 drop-out (%) | 12 (-14–33) | 3 (-15–24) | -63 (-163–-11) | 10 (-5–22) | 4 (-20–31) | -1 (-18–11) | -1 (-27–19) |
| Doses per fully immunized child | 14 (11–16) | 12 (8–13) | 9 (6–17) | 16 (11–27) | 14 (11–18) | 12 (7–20) | 14 (11–16) |
| Doses per full-time equivalent | 22 (12–28) | 25 (5–50) | 5 (2–12) | 14 (8–25) | 16 (10–31) | 36 (26–58) | 18 (10–39) |
| **Primary Health Centre** | | | | | | | |
| Sampled facilities | 7 | 18 | 12 | 13 | 16 | 6 | 17 |
| Target population | 11907 (5000–21000) | 5824 (2710–11591) | 5223 (2988–8922) | 8475 (3261–24624) | 8195 (5265–30045) | 7863 (5300–10788) | 8276 (3983–30000) |
| Target children (0–1 year) | 345 (150–630) | 129 (37–290) | 74 (31–162) | 273 (51–548) | 135 (74–487) | 229 (143–276) | 136 (54–315) |
| Doses administered | 3119 (1652–5320) | 1418 (300–3111) | 1572 (334–4928) | 2509 (682–4660) | 1780 (1025–6091) | 2467 (1837–2934) | 1818 (695–4203) |
| Sessions conducted (RI) | 127 (90–250) | 64 (24–107) | 54 (41–65) | 128 (32–237) | 60 (48–100) | 81 (68–97) | 37 (22–53) |
| Children vaccinated with DPT3/pentavalent3 | 229 (133–431) | 123 (34–256) | 185 (40–568) | 192 (43–373) | 127 (51–434) | 178 (117–227) | 131 (45–314) |
| Fully immunized children | 233 (123–406) | 119 (27–261) | 165 (37–473) | 167 (38–288) | 121 (66–366) | 198 (147–330) | 121 (46–291) |
| DPT3/pentavalent3 coverage rate (%) | 68 (49–91) | 98 (61–124) | 219 (129–376) | 75 (45–104) | 96 (63–138) | 74 (71–82) | 96 (60–117) |
| Full immunization coverage rate (%) | 70 (46–99) | 94 (73–136) | 202 (119–368) | 67 (39–88) | 95 (75–142) | 92 (68–120) | 89 (67–117) |
| DPT1 to DPT3/pentavalent1 to pentavalent3 drop-out (%) | 17 (-5–37) | -1 (-60–21) | -61 (-274–-1) | 8 (-12–23) | 4 (-34–41) | 6 (-9–17) | 0 (-23–17) |
| Doses per fully immunized child | 14 (11–16) | 12 (9–14) | 9 (8–11) | 15 (11–19) | 14 (12–18) | 13 (9–16) | 15 (12–18) |
| Doses per full-time equivalent | 28 (19–47) | 8 (3–18) | 7 (2–16) | 12 (4–29) | 16 (6–29) | 28 (22–41) | 16 (5–32) |

| | Bihar | Gujarat | Kerala | Meghalaya | Punjab | Uttar Pradesh | West Bengal |
|---|---|---|---|---|---|---|---|
| **Community Health Centre** | | | | | | | |
| Sampled facilities | 5 | 6 | 6 | 5 | 8 | 6 | 8 |
| Target population | NA | NA | 6379 (4585–8557) | 7609 (5457–11423) | 11638 (5566–22000) | 16362 (15157–17566) | 7463 (5354–9973) |
| Target children (0–1 year) | NA | NA | 74 (43–101) | 192 (94–322) | 196 (103–330) | 410 (380–439) | 115 (82–144) |
| Doses administered | 11126 (408–29907) | 1602 (283–3667) | 2112 (535–5794) | 2615 (1328–4659) | 3459 (957–7931) | 6493 (4195–10315) | 1894 (1232–2411) |
| Sessions conducted (RI) | 171 (89–285) | 50 (45–60) | 55 (50–62) | 134 (60–252) | 74 (44–116) | 187 (88–312) | 44 (36–60) |
| Children vaccinated with DPT3/pentavalent3 | 568 (30–1588) | 123 (3–309) | 245 (65–643) | 185 (90–362) | 203 (58–412) | 405 (221–655) | 130 (91–191) |
| Fully immunized children | 490 (21–1543) | 131 (3–307) | 214 (54–496) | 179 (93–341) | 200 (49–448) | 443 (236–750) | 137 (91–186) |
| DPT3/pentavalent3 coverage rate (%) | — | — | 301 (96–670) | 95 (70–117) | 99 (52–163) | 92 (77–107) | 111 (87–133) |
| Full immunization coverage rate (%) | — | — | 272 (79–517) | 93 (73–111) | 96 (44–177) | 121 (111–131) | 118 (87–171) |
| DPT1 to DPT3/pentavalent1 to pentavalent3 drop-out (%) | 13 (-25–53) | 13 (-17–35) | -50 (-81–-4) | 11 (-6–29) | 8 (-18–32) | 13 (-6–25) | 3 (-6–20) |
| Doses per fully immunized child | 23 (13–42) | 27 (10–94) | 9 (7–12) | 15 (14–17) | 18 (14–27) | 16 (9–22) | 14 (12–16) |
| Doses per full-time equivalent | 23 (2–44) | 29 (3–54) | 7 (2–16) | 12 (8–20) | 18 (7–33) | 26 (19–38) | 10 (6–14) |
| **District Hospital Post-partum Unit** | | | | | | | |
| Sampled facilities | 3 | 4 | 3 | 3 | 4 | 2 | 4 |
| Doses administered | 48202 (36840–67858) | 13122 (3073–23538) | 17128 (6991–23224) | 13502 (4472–26792) | 19054 (16039–22711) | 48934 (21703–76164) | 37212 (15879–55618) |
| Sessions conducted (RI) | 285 | 144 (96–240) | 101 (96–104) | 188 (50–285) | 175 (48–285) | 136 (104–168) | 158 (92–285) |
| Children vaccinated with DPT3/pentavalent3 | 2082 (1451–3296) | 448 (188–751) | 807 (368–1407) | 533 (223–734) | 1026 (290–1655) | 2259 (1177–3341) | 962 (138–2768) |
| Fully immunized children | 1880 (1304–2930) | 395 (141–672) | 695 (294–1254) | 443 (222–605) | 932 (263–1372) | 2380 (1055–3704) | 1020 (145–3104) |
| DPT1 to DPT3/pentavalent1 to pentavalent3 drop-out (%) | 29 (22–42) | 20 (12–39) | 33 (0–61) | 19 (11–34) | 14 (-2–32) | 24 (18–30) | 25 (8–43) |
| Doses per fully immunized child | 27(23–28) | 31 (22–35) | 28 (17–43) | 30 (15–53) | 29 (16–66) | 21 | 96 (15–212) |
| Doses per full-time equivalent | 115 (57–208) | 51 (17–71) | 34 (22–49) | 63 (39–90) | 91 (50–139) | 132 (103–161) | 99 (70–123) |

Notes: Average and figures in parentheses indicate range. NA indicates not available. Blanks indicate unable to calculate.
